# The Movement of Pathogen Carrying Flies at the Human–Wildlife Interface

**DOI:** 10.1007/s10393-022-01621-8

**Published:** 2023-01-11

**Authors:** Mueena Jahan, Sébastien Calvignac-Spencer, Colin A. Chapman, Urs Kalbitzer, Fabian H. Leendertz, Patrick A. Omeja, Dipto Sarkar, Markus Ulrich, Jan F. Gogarten

**Affiliations:** 1 Epidemiology of Highly Pathogenic Organisms, Robert Koch Institute, Berlin, Germany; 2 Department of Microbiology and Public Health, Bangabandhu Sheikh Mujibur Rahman Agricultural University, Gazipur, Bangladesh; 3 Viral Evolution, Robert Koch Institute Berlin, Berlin, Germany; 4 Wilson Center, 1300 Pennsylvania Avenue NW, Washington, DC USA; 5 Center for the Advanced Study of Human Paleobiology, George Washington University, Washington, DC USA; 6 School of Life Sciences, University of KwaZulu-Natal, Pietermaritzburg, South Africa; 7 Shaanxi Key Laboratory for Animal Conservation, Northwest University, Xi’an, China; 8 Department of Biology, University of Konstanz, Constance, Germany; 9 Department for the Ecology of Animal Societies, Max Planck Institute of Animal Behavior, Radolfzell, Germany; 10 Helmholtz Institute for One Health, Greifswald, Germany; 11 Makerere University Biological Field Station, Fort Portal, Uganda; 12 Department of Geography and Environmental Studies, Carleton University, Ottawa, Canada; 13 Present Address: Department of Applied Zoology and Nature Conservation, University of Greifswald, Greifswald, Germany

**Keywords:** Disease vector, disease risk, nonhuman primates, disease emergence

## Abstract

Flies form high-density associations with human settlements and groups of nonhuman primates and are implicated in transmitting pathogens. We investigate the movement of nonhuman primate-associated flies across landscapes surrounding Kibale National Park, Uganda, using a mark–recapture experiment. Flies were marked in nine nonhuman primate groups at the forest edge ($\overline{x}$ = 929 flies per group), and we then attempted to recapture them in more anthropized areas (50 m, 200 m and 500 m from where marked; 2–21 days after marking). Flies marked in nonhuman primate groups were recaptured in human areas (19/28,615 recaptured). Metabarcoding of the flies in nonhuman primate groups revealed the DNA of multiple eukaryotic primate parasites. Taken together, these results demonstrate the potential of flies to serve as vectors between nonhuman primates, livestock and humans at this biodiverse interface.

Synanthropic flies found in association with human settlements and their livestock have been implicated in the transmission of a large diversity of pathogens (Greenberg 1973). This includes bacteria [e.g., *Chlamydia trachomatis* (Forsey and Darougar 1981)], protozoan parasites [e.g., *Cryptosporidium parvum* (Clavel et al. 2002)], helminths [e.g., *Ascaris lumbricoides* (Adenusi and Adewoga 2013)], as well as viruses [e.g., turkey coronavirus (Calibeo-Hayes et al. 2003)]. Given this potential to serve as disease vectors, higher fly densities are associated with increased disease risk (Graczyk et al. 2001; Calibeo-Hayes et al. 2003). The synanthropic flies implicated in increasing disease risk encompass a taxonomically broad and extremely species rich group of Diptera, including a rich diversity from the families Calliphoridae, Sarcophagidae and Muscidae (Greenberg 1973; Stoffolano 2022).

Research suggests that such flies not only form associations with human and livestock populations, but also with wild nonhuman primate groups. For example, fly densities were higher in groups of sooty mangabeys (*Cercocebus atys*), chimpanzees (*Pan troglodytes*), baboons (*Papio anubis*), red colobus (*Piliocolobus tephrosceles*) and black-and-white colobus (*Colobus guereza*), than outside these groups (Gogarten et al. 2019, 2022). A mark–recapture experiment in a group of sooty mangabeys showed that flies can follow a group for up to 13 days, indicative of long-term associations (Gogarten et al. 2019). Particularly in species with a small home range and low daily travel distance, the density of flies in a group increased with larger group sizes (Gogarten et al. 2022). Much like their human-associated counterparts, nonhuman primate-associated flies include a rich diversity of species from the families Calliphoridae, Sarcophagidae and Muscidae (Gogarten et al. 2019, 2022). Collectively, this research suggests that flies form relatively stable associations with a wide range of nonhuman primate species.

These nonhuman primate-associated flies can also carry pathogens and likely increase disease risk. For example, flies associated with a group of sooty mangabeys carried viable *Bacillus cereus* biovar *anthracis*, which causes sylvatic anthrax (Hoffmann et al. 2017; Gogarten et al. 2019). Flies in this group of sooty mangabeys also contained the DNA of *Treponema pallidum pertenue* (Gogarten et al. 2019), which causes yaws disease, which was described in flies from another ecosystems as well (Knauf et al. 2016). Synanthropic flies have been implicated in yaws transmission (Lamboen 1936; Barnard 1952), though it remains unclear to what extent nonhuman primate-associated flies are really involved in the transmission of *Treponema pallidum pertenue* or *Bacillus cereus* biovar *anthracis*. Both *Bacillus cereus* biovar *anthracis* and *Treponema pallidum pertenue* were detected in broad diversity of nonhuman primate-associated fly species; in a subset of 96 flies captured in a group of sooty mangabeys that included 14 putative species, viable *Bacillus cereus* biovar *anthracis* was detected in two fly species, while *Treponema pallidum pertenue* DNA was detected in four other species. Collectively, this suggests that high densities of nonhuman primate-associated flies may pose an increased disease risk by increasing within-group transmission and contamination of substrates that animals come into contact with (Gogarten et al. 2022), though the range of pathogens explored to date remains extremely limited.

The detection of duiker DNA in flies in a sooty mangabey group, as well as the detection of a fly marked in this sooty mangabey group in a chimpanzee group, suggests flies can transfer between animal species and could play a role in between-species transmission as well (Gogarten et al. 2019). This echoes the finding that synanthropic flies can serve as vectors between livestock and humans (Rosef and Kapperud 1983; Khamesipour et al. 2018). Thus, a critical question for nonhuman primate-associated flies at the human wildlife interface is their potential to transmit infectious agents from nonhuman primates (and other wildlife) to livestock and humans. This can be addressed in part by investigating the stability of these associations and the mobility of these flies. A small-scale analysis of the mammalian DNA found in flies in a village near Taï National Park detected the DNA of wildlife species, which could be considered evidence that forest flies enter human habitats (Gogarten et al. 2019). Another possibility though, is that these flies were exposed to the DNA of larger mammal species’ through contact with bushmeat in villages, as both duikers and colobines are frequently hunted in this region, while the two rodent species detected are often found in and near human habitats (Refisch and Koné 2005).

Here, we conduct a mark–recapture experiment at the forest edge of Kibale National Park, Uganda to determine whether flies move between wildlife and human populations. We marked flies in nine nonhuman primate groups at the forest edge and tried to recapture these flies in more anthropized areas. To explore the potential disease risk that the movement of nonhuman primate-associated flies into anthropized areas poses, we screened nonhuman primate-associated flies for eukaryotic primate parasites using insect soup metabarcoding (Yu et al. 2012). Kibale National Park consists of a mid-elevation semievergreen forest and contains 13 species of nonhuman primates. We marked 8365 flies in nine groups of four nonhuman primate species that are frequently found at the forest edge close to human settlements (Fig. 1): black-and-white colobus, red colobus, gray-cheeked mangabeys (*Lophocebus albigena*) and red-tailed guenons (*Cercopithecus ascanius*). Nonhuman primate groups were selected opportunistically when they were detected at the forest edge and the number of flies marked was determined by the fly capture rate once a nonhuman primate group was detected.

**Figure 1:**
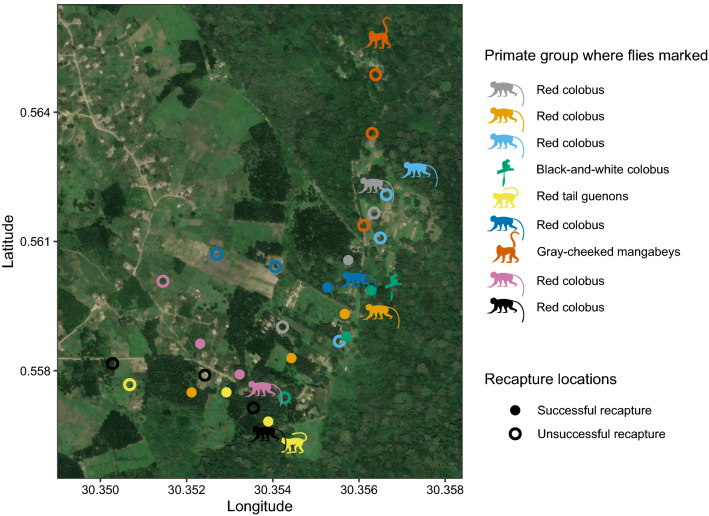
Locations where flies were marked in primate groups and where recapture effort in more anthropized areas occurred. Monkey silhouettes of different colors indicate the group location at the forest edge where flies were marked on a specific day. Circles indicate sites where recapture effort was targeted on subsequent days. Solid filled circles indicate a location where a marked fly was recaptured, while an unfilled circle indicates no flies were recaptured at that location. Colors of circles correspond to recapture effort toward flies marked on a specific date, indicated by the colors of the monkey silhouettes (Color figure online).

Flies were captured using custom-made traps (described in: Hoffmann et al. 2017) placed over a commercial attractant based on animal proteins that mimic a decaying carcass (Unkonventionelle Produkte Feldner, Waldsee, Germany) or a piece of day-old chicken or beef. Flies were marked in large batches in a plastic bag with Glo-Germ powder (Glo Germ Co., Moab, Utah), which can be visualized under UV light. All flies captured during the initial capture event in the nonhuman primate group were marked with powder. Between any two consecutive months of the experiment, we used a different powder color to mark flies in the nonhuman primate groups to avoid incorrect assignment of where flies were marked. To explore fly mobility in anthropized areas, we attempted to recapture flies in the human habitat at a distance of roughly 50 m, 200 m and 500 m from where they were initially marked. Recapture attempts occurred 2, 4, 7, 14 and 21 days after marking, resulting in a total effort of 28,615 flies, with recapture effort dictated by the capture rate at these locations. Flies were checked for Glo Germ powder with a UV light. Kibale National Park is characterized by two rainy and two dry seasons and to explore potential seasonal variation in fly mobility, we compare the monthly rainfall totals assessed immediately adjacent to the study area in months during which recapture occurred and those in which no recapture events occurred (Chapman et al. 2021).

A total of 19 of the 8365 marked flies (0.23%) were recaptured away from the nonhuman primate group in anthropized areas (Fig. 1; Table 1). This included 9 flies at a distance of 50 m (recapture effort = 9681 flies), 8 flies at a distance of 200 m (recapture effort = 9937 flies) and 2 flies at a distance of 500 m (recapture effort = 8997 flies) from where they were marked. Flies marked in groups of three of four species of nonhuman primate examined were recaptured outside of these groups (all except from the gray-cheeked mangabey group). Compared to these recapture rates in anthropized areas, a mark–recapture experiment in a mangabey group in Taï National Park, Côte d'Ivoire found a much higher recapture rate (51/1591 = 3.2% of marked flies recaptured; recapture effort = 3164; Gogarten et al. 2019). Collectively these findings suggests that flies preferentially maintain an association with a nonhuman primate social group and maintain these associations, but do occasionally leave these association and move into anthropized areas.

**Table 1:** Number of flies marked and recaptured in six nonhuman primate groups.

Mark date	Primate species	Distance of trap location	N flies marked in primate group	N flies recapture effort	N marked flies recaptured
10/24/20	Red colobus	In group	396		
		50 m		936	0
		200 m		1298	1
		500 m		1324	0
11/20/20	Red colobus	In group	435		
		50 m		982	2
		200 m		1211	3
		500 m		1445	2
1/15/21	Red colobus	In group	580		
		50 m		1517	0
		200 m		1080	0
		500 m		1782	0
2/11/21	Black-and-white colobus	In group	514		
		50 m		1145	2
		200 m		974	1
		500 m		1034	0
3/11/21	Red-tailed guenons	In group	955		
		50 m		2026	1
		200 m		1735	2
		500 m		990	0
4/8/21	Red colobus	In group	1485		
		50 m		1370	3
		200 m		1515	0
		500 m		422	0
5/6/21	Gray-cheeked mangabeys	In group	1270		
		50 m		515	0
		200 m		584	0
		500 m		530	0
6/3/21	Red colobus	In group	1550		
		50 m		840	1
		200 m		1080	1
		500 m		1130	0
7/3/21	Red colobus	In group	1180		
		50 m		350	0
		200 m		460	0
		500 m		340	0
		Total	8365	28,615	19

Recapture rates declined with increasing time since flies were marked; 12 flies were recaptured 2 days after marking (recapture effort = 6117 flies) and 7 flies after 4 days (recapture effort = 6469 flies), while no flies were recaptured after 7 days (recapture effort = 4450 flies), after 14 days (recapture effort = 5777 flies) or after 21 days (recapture effort = 5902 flies). The time dependent decay in recapture success may suggest either diffusion of flies, the loss of marking powder through time or relatively short fly survival post-capture (due to the combination of short lifespans and age at capture, capture stress or exposure to the powder), hypotheses that we are not able to differentiate further here. While the small sample sizes preclude a robust statistical analysis of seasonality on recapture rates, those months during which no recapture events occurred were drier months (Fig. 2). This might suggest that fly mobility, survival or loss of marking powder is influenced by rainfall or other climatic factors, but future studies with larger samples sizes across different seasons are needed to rigorously explore the importance of these factors.

**Figure 2:**
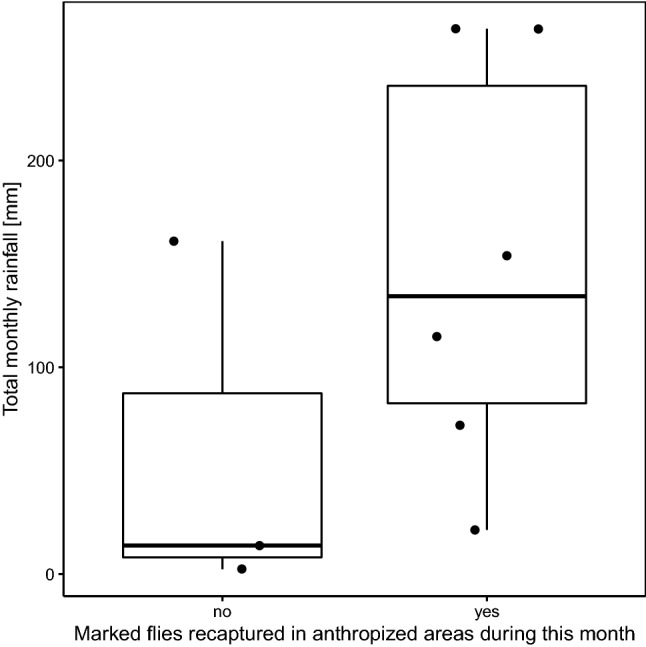
Box-and-whisker plot of monthly rainfall totals at the study site during months during which a recapture event occurred in the anthropized areas, or a recapture event did not occur. Lower and upper hinges correspond to the 25th and 75th percentiles, respectively, while the middle horizontal line represents the median. The upper and lower whiskers extend to the largest and lowest values no more than 1.5 times the interquartile range away from the hinge. Raw data are plotted in solid points.

We previously determined the fly species present inside the social groups of nonhuman primates in Kibale using soup metabarcoding of a fragment of the mitochondrial gene, cytochrome oxidase C subunit 1 (COI), detecting flies in the families Calliphoridae, Sarcophagidae and Muscidae (Gogarten et al. 2022). In these previous experiments, we removed a leg from flies captured in groups of six nonhuman primate species (*N* = 575 fly legs) and legs were pooled by nonhuman primate species and homogenized with a Tissuelyser II (Qiagen) and DNA extracted with the GeneMATRIX Stool DNA Purification Kit (Roboklon). To explore whether the same fly species were present outside nonhuman primate groups, we had also homogenized fly legs from the same flies captured 500 m outside groups (*N*_files_ = 575, *N*_pools_ = 6) and included a pool of 100 fly legs from flies captured in the Volkspark Rehberge, Berlin, Germany and an extraction blank as controls.

To explore the potential disease risk posed by the movement of flies from primate groups into anthropized, we molecularly characterized the eukaryotic parasites detected on flies captured in nonhuman primate groups. We performed soup metabarcoding to detect eukaryotic parasites, using the same pools of fly-leg extracts described above (for details of the extraction methods, see: Gogarten et al. 2022). Specifically, we applied a PCR system targeting the 18S rRNA of eukaryotic parasites (methods described in detail here: Maritz et al. 2017; Amaral-Zettler et al. 2018); we modified the protocol by using the two universal primers Euk 1391F and EukBr with nextera specific overhangs to amplify the V9 variable region of the 18S rRNA of eukaryotic parasites (Gohl et al. 2016). We then prepare amplicons for sequencing with a second PCR to append sequencing adapters and sample specific indexes. We included three negative controls and include one extraction blank with the PCR. Cycling conditions were 98°C for 5 min, 25 cycles of 98°C for 20 s, 65°C for 15 s, 57°C for 30 s and a final step of 72°C for 10 min (modified from Maritz et al. (2017). Products were visualized on 1.5% agarose gels and cleaned using AMPure XP Beads and pools uniquely dual indexed using the Nextera XT Index kit and sequenced on an Illumina NextSeq 500 with a mid-output kit v.2 and 2 × 150 cycles.

We removed primers using cutadapt (v 2.1: Martin 2011) and filtered reads using the DADA2 pipeline (Callahan et al. 2016) and assigned them taxonomically using the RDP naïve Bayesian classifier algorithm coupled with the PR2 training database (v 4.12.0: Wang et al. 2007). Poor read quality for the second read precluded its use in the analysis. To consider only parasites relevant to primate health, we considered reads assigned to families that include known primate parasites (following: Gogarten et al. 2020). We did not identify any such read in the negative controls (i.e., the extraction blank, the no template controls or the Berlin flies), but we detected reads belonging to three families of eukaryotic primate parasites in the flies captured in primate groups: Blastocystidae, Entamoebidae and Vahlkampfiidae (Fig. 3). In addition, from flies captured within the forest but outside primate groups, we detected parasites belonging to the same parasite families and the family Trypanosomatidae (no reads from flies of the Glossina genus were detected in the soup metabarcoding effort describing this fly community; Figure 3; Gogarten et al. 2022). While we here described the parasites carried by nonhuman primate-associated flies in Kibale, a clear limitation is that we did not collect and export the 19 primate-associated flies that we recaptured in anthropized areas, which precluded a determination of their species or the parasites these particular flies carried. This is an important area of future research and we encourage future research to explore which particular nonhuman primate-associated fly species tend to move into anthropized areas and the particular disease risk these specific fly species pose. Future research could provide an in-depth understanding of the individual fly species carrying particular pathogens and these species’ particular behavior and interactions with primates that the current study could not provide. Furthermore, the detection of parasite DNA does not prove infectivity of these parasites and future studies are needed to explore the actual disease risk posed by the pathogens found in and on nonhuman primate-associated flies.

**Figure 3:**
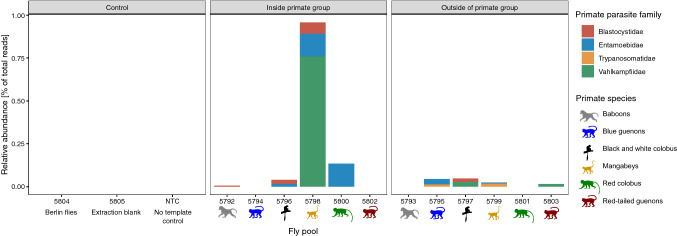
Relative abundance of primate parasite families detected in flies captured inside and outside primate groups and for the control experiments. Monkey silhouettes of different colors indicate the primate species from which flies were collected. The color of the solid bars indicates the primate parasite family detected. Numbers correspond the fly pool extract on which soup metabarcoding was performed (details available in: Gogarten et al. 2022) (Color figure online).

Vector-borne diseases appear to be emerging at an increasing rate, comprising a disproportionate share of emerging infectious diseases, particularly in Africa (Swei et al. 2020). The close evolutionary relationship and resultant similar physiology of nonhuman primates and humans make nonhuman primates a likely source for the zoonotic transmission of pathogens (Gillespie et al. 2008; Calvignac-Spencer et al. 2012, 2021). At the same time, human pathogens have shown their potential to cause mortality in nonhuman primate populations (Köndgen et al. 2008). Areas of between-species transmission are predicted to be highest around the forests of central and west Africa, where humans often come into contact with wild primates; contact between wildlife and humans is expected to rise as human populations continue to grow and habitat fragmentation increases (Pedersen and Davies 2009). Early studies of *Escherichia coli* bacteria in humans and primates in anthropically disturbed areas at the forest edge of Kibale National Park may suggest regular bidirectional, interspecific bacterial transmission (Goldberg et al. 2008), though more rigorous methods (e.g., phylogenomic analyses) are needed to confirm this hypothesis. With this study, we suggest that flies, which serve as mechanical vectors for infectious agents, require further consideration as vectors between human and wildlife populations. Understanding factors that facilitate the movement of flies across the human–wildlife interface may ultimately enable the implementation of mitigation strategies such as the construction and donation of latrines to people living on the edges of parks.

## Data Availability

Metabarcoding sequencing data are available as a Zenodo dataset: 10.5281/zenodo.7509929.
